# The Future of Obesity Care: Exploring Synergies Between Metabolic Bariatric Surgery, Interventional Endoscopy and Pharmacotherapy

**DOI:** 10.1007/s11695-026-08692-4

**Published:** 2026-05-04

**Authors:** Wendy A. Brown, Priya Sumithran, Michael Cowley, Ricardo Cohen, Carel Le Roux, Philip Schauer, Santosh Agarwal, Jamie Ard, Camilo Boza, Jenny Lynn Cargiuolo, Letizia Ceccarelli, Jake Coverdale, Brian Dunkin, Francis Finucane, Kapil Gupta, Lee M. Kaplan, Marina Kurian, Ildiko Lingvay, Alex Miras, Chuck Pearlman, Terissa Petry, Dimitri Pournaras, Jonthan Purnell, Roxana Ruiz, Carlos Schiavon, Abd Tahrani, Hanan Tarek, Jorg Tomaszewski, Sascha Tumik, Ramy Younes

**Affiliations:** 1 Department of Surgery, Monash University, Melbourne, Australia; 2 Oesophgago-Gastric and Bariatric Unit, Alfred Health, Melbourne, Australia; 3 Obesity and Metabolic Medicine Group, Alfred Health, Melbourne, Australia; 4 Biomedicine Discovery Institute, Monash University, Melbourne, Australia; 5 Obesity and Diabetes Center, Hospital Alemão Oswaldo Cruz, São Paulo, Brazil; 6 University College Dublin, Dublin, Ireland; 7 Pennington Biomedical Research Center, Baton Rouge, United States; 8 Medtronic (United States), Minneapolis, United States; 9 School of Medicine, Wake Forest University, Winston-Salem, United States; 10 Center for Nutrition and Bariatrics, Clínica Las Condes, Santiago, Chile; 11 Boston Scientific (United States), Marlborough, United States; 12 W. L. Gore & Associates (United States), Newark, United States; 13 University of Galway, Galway, Ireland; 14 University Hospital Galway, Galway, Ireland; 15 Geisel School of Medicine, Dartmouth College, Hanover, United States; 16 NYU Langone Hospital - Long Island, Mineola, United States; 17 Department of Internal Medicine/Division of Endocrinology, The University of Texas Southwestern Medical Center, Dallas, United States; 18 Department of Medicine, University of Ulster, Coleraine, United Kingdom; 19 Department of Surgery, North Bristol NHS Trust, Bristol, United Kingdom; 20 Knight Cardiovascular Institute, School of Medicine, Oregon Health & Science University, Portland, United States; 21 Center of Obesity and Metabolic Disorders, Sao Paulo, Brazil; 22 Novo Nordisk (Denmark), Copenhagen, Denmark; 23 Eli Lilly (Australia), Sydney, Australia; 24 Johnson & Johnson (United States), New Brunswick, United States; 25 Boehringer Ingelheim (Germany), Ingelheim am Rhein, Germany

## Abstract

With the advent of new effective treatments for obesity, the field is rapidly changing creating an urgent need for evidence to guide best patient management. To help prioritise and plan for future trials, a meeting of experts was convened with the aim of reviewing the current literature to identify and prioritise current knowledge gaps; identify relevant research questions, and discuss appropriate trial methodologies that could be utilised to address the identified gaps in a timely and pragmatic manner. Participants included research-active academic surgeons and physicians, and industry representatives from various pharmaceutical and device companies. This report summarizes the key outcomes from this meeting.

## Key Points

Treatment options for obesity are rapidly evolving.

To provide best personalised care for patients, more evidence is required to understand how to best utilise currently available treatments as well as combine treatments.

Trials focused on improving the treatment of obesity may need to be pragmatic and more agile than the traditional RCT to enable real time impact on patient care.

## Introduction

The regulatory approval of the glucagon-like peptide-1 receptor agonist (GLP-1RA) semaglutide 2.4 mg for chronic weight management in 2021 marked the beginning of a new treatment era for obesity. Tirzepatide, a dual agonist at GLP-1 and glucose-dependent insulinotropic polypeptide (GIP) receptors was approved for obesity treatment in 2023. Several more promising medications such as cagrisema [1], survodutide [2], and retatrutide [3] are already in phase 3 regulatory studies, and many more in earlier phases of development [3, 4]. This new generation of obesity medications results in mean weight loss of 15–25% over 68–72 weeks in clinical trials, narrowing the efficacy gap between medical and surgical management of obesity [5, 6].

In chronic disease management, it is standard practice to use complementary treatment options sequentially or in combination, adapting the treatment plan over the disease course. As such, it is inevitable that for some people with obesity, the optimal treatment plan will include, concomitantly or sequentially, obesity management medications (OMM) and metabolic bariatric surgical (MBS) or endoscopic procedures.

Recently, a consensus statement on the role of OMM in the context of MBS was developed by the International Federation for the Surgery of Obesity and Metabolic Disorders (IFSO) [7]. Acknowledging the scarcity of data on which to base their recommendations, the consensus statement highlighted areas warranting future research. The emergence of effective endoscopic therapies for obesity [8–10 also warrants consideration of how they may be integrated into the treatment pathway to improve outcomes or address complications.

To better anticipate and delineate high-priority investigatory needs concerning the combined use of medications, endoscopic and surgical therapies for obesity, we convened a multidisciplinary workshop from August 30 – to September 1, 2024. Our expert group included research-active academic surgeons and physicians, and industry representatives from various pharmaceutical and device companies (Table 1). The workshop aims were to (1) identify knowledge gaps after considering the current literature, (2) identify relevant research questions, and (3) discuss appropriate trial methodologies that could be utilised to address the identified gaps in a timely and pragmatic manner.

**Table 1:** List of participants

Name	Position	Company	Conflicts of Interest
Agarwal, Santosh	Senior Director Evidence & Economics	Medtronic	Employee and shareholder of Medtronic
Ard, Jamy	Vice Dean for Clinical Research	Wake Forest School of Medicine, North Carolina, USA	Research Support - Nestle Healthcare Nutrition, Eli Lilly, Boehringer Ingelheim, Epitomee, Inc., United Health Group, R&D, KVKTech, WW, Novo Nordisk, Regeneron, Amgen Consulting and Advisory Board- Nestle Healthcare Nutrition, Eli Lilly, Novo Nordisk, Regeneron, Amgen, Ingredion, Zealand Pharma, Almond Board Memberships-International Food Information Council- Assembly, The Obesity Society- President 2024, Roundtable on Obesity Solutions, American Society for Nutrition, American Society for Nutrition Foundation- Board of Trustees Executive Committee
Boza, Camilo	Bariatric Surgeon and Medical Director	Center for Nutrition and Bariatrics, Clinica Las Condes, Chile	Johnson & Johnson Medtech: Consultant Boston Scientific : Consultant GT Metabolic : Research Grant
Brown, Wendy*	Professor of Surgery and Director of Surgical Services	Monash University, Alfred Health, Australia	Grants from Johnson and Johnson, Medtronic, GORE, Applied Medical, Novo Nordisk, NHMRC, Myerton and the Australian Commonwealth Government. Personal fees from Johnson and Johnson, GORE, Novo Nordisk, Pfizer, Medtronic, Lily and Merck Sharpe and Dohme for lectures and advisory boards.
Cargiuolo, Jenny Lynn	Senior Manager, Global Obesity Metabolic Health Market Development	Medtronic	Employee of Medtronic
Ceccarelli, Letizia	Assoc. Director International EB Marketing	Boston Scientific	Employee and shareholder of Boston Scientific
Cohen, Ricardo*	Director, Obesity and Diabetes Centre	Hospital Alemao Oswaldo Cruz, Brazil	declares research grants from Johnson & Johnson, Marlex Brasil and Medtronic; honoraria for lectures and presentations from Johnson & Johnson, Medtronic, Boston Scientific, Merck and NovoNordisk; and serving on scientific advisory boards for Morphic Medical, Johnson & Johnson, Boston Scientific, Regeneron and Medtronic
Coverdale, Jake	ANZ Medical Business Leader-Medical Products Division	W.L. Gore	Employee of W.L Gore & Associates
Cowley, Michael*	Professor of Physiology	Biomedicine Discovery Institute, Monash University, Australia	Grants from Novo Nordisk Consulting fees from Zealand Pharmaceuticals, iNova, Novo Nordisk.
Dunkin, Brian	Chief Medical Officer, VP Medical Affairs	Boston Scientific	Employee and shareholder of Boston Scientific
Finucane, Francis	Endocrinologist and Honorary Full Professor of Medicine	University of Galway and Galway University Hospitals, Gallway, Ireland	Grants from Saolta University Healthcare Group and Science Foundation Ireland. Paid DSMB member for LEAP and LEGEND RCTs at the University of Michigan. Unpaid Principal Investigator on REDEFINE 2 and REDEFINE 3 RCTs with Novo Nordisk. Board Member, Irish Heart Foundation.
Gupta, Kapil	VP of Clinical and Scientific Affairs, BSC Endoscopy (Global)	Boston Scientific	Employee and shareholder of Boston Scientific
Kaplan, Lee M.	Professor of Medicine	Geisel School of Medicine at Dartmouth, USA	Consultant to Altimmune, Amgen, AstraZeneca, Boehringer Ingelheim, Cytoki, Ethicon, Gilead Sciences, Helicore, Intellihealth, Johnson & Johnson, Kallyope, Eli Lilly & Company, Metsera, MetaVia, Neurogastrx, Novo Nordisk, Optum Health, Oxford Medical Products, Perspectum, Pfizer, Rhythm Pharmaceuticals, Skye Bioscience, Structure Therapeutics, The Last Food Fight and twenty30.health.
Kurian, Marina	Bariatric Surgeon	NYU, Langone Health, USA	Honoraria for speaking: Medtronic, Ethicon, Stryker, Novo Nordisk
Le Roux, Carel*	Professor of Chemical Pathology	University College Dublin, Ireland	Grants from the EU Innovative Medicine Initiative, Irish Research Council, Science Foundation Ireland, Anabio, and the Health Research Board. Advisory boards and speakers panels of Novo Nordisk, Roche, Herbalife, GI Dynamics, Eli Lilly, Johnson & Johnson, Gila, Irish Life Health, Boehringer Ingelheim, Currax, Zealand Pharma, Keyron, AstraZeneca, Arrowhead Pharma, Amgen, AbbVie, Metsera, Nymble, Olympus, and Rhythm Pharma. Received stock options as payment for scientific advisory board functions from Metsera and Nymble. Provides obesity clinical care in the My Best Weight clinic and Beyond BMI clinic and is a co-owner of these clinics.
Lingvay, Ildiko	Tenured Professor of Medicine Department of Internal Medicine/Division of Endocrinology Peter O’Donnel Jr. School of Public Health Medical Director, Office of Clinical Trials Management Executive Director, Diabetes and Obesity Research Program	University of Texas Southwestern Medical Center, Dallas, TX, USA	received research funding (paid to institution) and/or product from NovoNordisk, Boehringer-Ingelheim, Dexcom, Roche, Pfizer, Lilly. IL received research related consulting fees (paid to institution) from NovoNordisk. IL received advisory/consulting fees and/or other support from: Abbvie, Altimmune, Alveus Therapeutics, Amgen, Antag Therapeutics, Astra Zeneca, Bain Capital, Bayer, Betagenon AB, Bioio Inc., Biomea, Boehringer-Ingelheim, Carmot, Corxel, Cytoki Pharma, Eli Lilly, Genentech, Intercept, Janssen/J&J, Juvena, Keros Therapeutic, Inc, Mediflix, Merck, Metsera, Neurocrine, Novo Nordisk, Pfizer, Regeneron, Roche, Sanofi, Shionogi, Source Bio, Structure Therapeutics, TERNS Pharma, The Comm Group, Verdiva Bio, WebMD, and Zealand Pharma.
Miras, Alex	Clinical Professor of Medicine	Ulster University, UL	has received research funding from the European Union, Medical Research Council (MRC), National Institute for Health and Care Research (NIHR), HSC R&D division, Jon Moulton Charitable Foundation, Anabio, Fractyl, Boehringer Ingelheim, Eli Lilly, Gila, Randox, and Novo Nordisk. ADM has received honoraria for lectures and presentations from Novo Nordisk, AstraZeneca, Currax Pharmaceuticals, Boehringer Ingelheim, Screen Health, GI Dynamics, Algorithm, Eli Lilly, Ethicon, and Medtronic. ADM is a shareholder in the Beyond BMI clinic, which provides clinical obesity care.
Pearlman, Chuck	Sr Director of Marketing, Endoluminal Surgery	Boston Scientific	Employee and shareholder of Boston Scientific
Petry, Tarissa	Endocrinologist	Hospital Alemao Oswaldo Cruz, Brazil	Research grant- Medtronic. Speaker for NovoNordisk, Merck, Johnson&Johnson Medtech, Medtronic
Pournaras, Dimitri	Bariatric Surgeon	North Bristol Trust, UK	has been funded by the Royal College of Surgeons of England. He receives consulting fees from GSK, Johnson & Johnson, Novo Nordisk, Eli-Lilly and Boston Scientific and payments for lectures, presentations and educational events from Johnson & Johnson, Medtronic, Novo Nordisk, Pfizer and Sandoz.
Purnell, Jonathan	Professor of Medicine	Knight Cardiovascular Institute, School of Medicine, Oregon Health & Science University, USA	Advisory boards of Novo Nordisk, Boehringer Ingelheim, Regeneron, and Zealand Pharma.
Ruiz, Roxana	EA to Wendy Brown	Monash University, Alfred Health, Australia	No conflicts
Schauer, Phil*	United Companies Life Insurance/Mary Kay and Terrell Brown Chair Professor	Pennington Biomedical Research Centre, Louisiana State University	reports receiving research grants from the National Institutes of Health, Ethicon and Medtronic; receiving personal consulting fees or honoraria from GI Dynamics; Keyron; Mediflix, Metabolic Health International, LTD, Heron, Novo Nordisk, Klens; serving on scientific advisory boards for SE Healthcare Board of Directors; Lilly, GI Dynamics; Keyron; Regeneron; Mediflix, and having ownership interest in SE Healthcare LLC, Mediflix, Metabolic Health International, LTD
Schiavon, Carlos	Bariatric Surgeon	Center of Obesity and Metabolic Disorders, Sao Paulo, Brazil	Research grant from Ethicon Inc. Honoraria from Johnson & Johnson Brasil
Sumithran, Priya*	Head Obesity and Metabolic Medicine Group	Monash University, Alfred Health, Australia	Co-authored manuscripts with a medical writer provided by Novo Nordisk and Eli Lilly and received payment to her academic institution from Novo Nordisk and Eli Lilly for speaking and advisory activities
Tahrani, Abd	International Medical Vice President, Medical & Science, Obesity and NASH, Clinical Drug Development	Novo Nordisk	was an employee of Novo Nordisk at the time of authoring the manuscript. He is now employed by Amgen Research Copenhagen. Amgen Research Copenhagen had no role in this manuscript.
Tarek, Hanan	Medical Team Australia	Eli Lilly	was an employee of Eli Lilly at the time of authoring the manuscript.
Tomaszewski, Jorg	Global Franchise Medical Director	Johnson and Johnson	Employee and shareholder of Johnson & Johnson
Tumik, Sascha	Snr. Market Development Manager ANZ, Surgical Innovations, Obesity & Metabolic Health	Medtronic	was an employee of Medtronic at the time of authoring the manuscript
Younes, Ramy	Global Head of Clinical Development Obesity & Liver Health	Boehringher-Ingelheim	Employee of Boehringer Ingelheim

*Denotes Steering Committee Member

### Gaps in our Understanding of Combining Obesity Therapies

#### Themes Identified through the IFSO Consensus Statement on Combining OMM and MBS

The group considered the recommendations of the IFSO consensus meeting [7] that identified areas where future research was needed (summarised in Table 2).

**Table:** 

Key themes identified as needing further research by IFSO [7]:
• The balance of improved weight loss and health outcomes compared with the risk of combining OMM with MBS (recommendations 6, 11, 12, 22)
• The ability to predict who is most likely to benefit from combining OMM and MBS (recommendation 10)
• The best dosing paradigm for OMM when combined with MBS and how this might be adjusted according to the goals of treatment (Recommendation 15, 23)

**Table 2:** Selected Research Priorities identified at the IFSO Consensus meeting on the use of OMM and MBS

Recommendation 4	There is insufficient high-level evidence to recommend the routine use of OMMs for weight loss before MBS
Recommendation 6	Future research is needed to explore the value of using OMMs before MBS to assess their benefits, risks, and clinical outcomes (A+; 100% agreement; 2 rounds of voting)
Recommendation 10	Future research is needed to identify predictors of which patients are likely to derive substantial benefit from combined pharmaco-surgical therapy for obesity and its complications (A+; 100% consensus, 3 rounds of voting)
Recommendation 11	MBS is strongly associated with reduced adverse cardiovascular events, and GLP1RA agonists have been shown to reduce such events. Future research is required to determine the benefits of combination treatment for these outcomes (A+; 100% consensus, 3 rounds of voting)
Recommendation 12	Both MBS and GLP1RA agonists reduce chronic kidney disease. Future research is required to determine the benefits of combination treatment for these outcomes (A+; 100% consensus, 3 rounds of voting)
Recommendation 15	Research on the intermittent use of OMMs and/or their dose adjustment after MBS with a suboptimal response is needed (A; 94% consensus; 3 rounds of voting)
Recommendation 21	As the long-term efficacy and safety of OMMs after MBS is unknown, studies are needed to understand the value and limitations of such combination therapy (A+; 100% consensus, 3 rounds of voting)
Recommendation 22	Endpoints of future clinical trials of existing and/or novel obesity-management interventions (behavioral, pharmacological, endoscopic, and surgical) should focus on improvement, remission, and prevention of clinical manifestations and complications of obesity in addition to weight loss (A+; 100% consensus, 3 rounds of voting)
Recommendation 23	Studies are needed to define stage-specific therapeutic protocols that integrate surgical intervention and adjuvant pharmacotherapy to achieve improvement (or remission when possible) of clinical obesity (A; 95% consensus; 1 round of voting pre-meeting)

#### Summary of Previous Studies Combining OM with MBS

Only a small number of randomised controlled trials (RCTs) have previously examined combination treatment with OMM and MBS. The first to study the effect of treatment with OMM after MBS measured the effect of liraglutide 1.8 mg on HbA1c reduction in participants with type 2 diabetes undergoing MBS who had persistent HbA1c levels higher than 48 mmol/mol (6·5%) at least one year after either sleeve gastrectomy or Roux-en-Y gastric bypass (GRAVITAS trial). After 26 weeks, liraglutide treatment was associated with a difference of −13·3 mmol/mol (−1·2%, 95% CI −19·7 to −7·0; *p* = 0·0001) in HbA1c change from baseline when compared with placebo. The estimated mean difference in weight change from baseline to week 26 for liraglutide versus placebo was − 4·2 kg (95% CI − 6·8 to − 1·6, *p* = 0·0017). The most reported side effects were gastrointestinal [11].

The BARI-OPTIMISE RCT aimed to assess the efficacy and safety of liraglutide 3.0 mg daily, the dose approved for weight management, on percentage body weight reduction in patients with sub-optimal weight loss and suboptimal nutrient-stimulated endogenous GLP-1 response after MBS. The mean difference in percentage body weight change at 24 weeks between liraglutide 3.0 mg and placebo was − 8.0% (95% CI, −10.4 to −5.7%; *P* <.001). Gastrointestinal side effects were the most reported adverse events [12].

Considering the available data on combination therapy, several further themes were identified as requiring further research.

**Table:** 

Key themes identified as requiring further research from prior RCT [11, 12]:
• Acceptability to patients of combination therapy.
• Longer term efficacy and safety of OMM, as well as OMM and MBS combinations.
• Cost effectiveness of combination therapy.
• Evidence to support choice of medication and treatment goals when commencing an OMM post-MBS.
• Optimal dosing of OMM and if there is need for long-term chronic therapy.
• Impact of combination therapy (including side effects) on Quality of Life.

#### Themes Identified through Expert Discussion

The group then considered the patient’s journey through MBS and where OMM or endoscopic therapies may be included in the paradigm of care:

1. *Qualifying criteria for MBS*: Currently, people living with obesity who present for consideration of MBS are assessed using the indications provided by the IFSO/ASMBS guidelines for MBS [13]. Guidance was considered to be lacking regarding the use of OMM or endoscopic therapies prior to MBS, particularly in specific populations:
  2. Older people: where the risks of surgery are higher and the possibility of improved associated health conditions is lower.
  3. Younger people: where lifelong care for obesity is anticipated.
  4. Lower BMI: where less weight loss is required to improve health but benefit from regional (i.e., visceral fat) loss may still be important.
2. *Preoperative preparation for MBS*: Retrospective studies suggest that modest weight loss (0–5% TBWL) prior to MBS, especially in patients with a high BMI (BMI > 50 kg/m2) may reduce the risk of death following MBS [14] as well as reducing post-operative complications [15, 16]. This is most likely due to weight loss reducing visceral adiposity and liver size, improving the surgical conditions [17], although the reports of intraoperative outcomes have been inconsistent in the literature. No level 1 evidence exists addressing the risk and benefit of routine weight loss before bariatric surgery, although 83% of centres in the U.S. engage in some type of preoperative weight loss effort with caloric restriction or OMM [15, 16]. Whilst caloric restriction has been the mainstay of all pre-MBS weight loss interventions, there is currently limited evidence supporting the best approach or duration of treatment. There is limited evidence for pre-operative use of older pharmaceutical agents [7], GLP-1 RA’s [18] and endoscopically placed intragastric balloons [19]. It was also noted that there is a need to better understand the timing of cessation of OMM prior to MBS [20, 21.
3. *Reassessment for MBS*: Greater than 20% TBWL was achieved by one third of participants treated with semaglutide 2.4 mg weekly in the STEP-1 trial [6] and by more than half of those treated with tirzepatide 15 mg weekly in the SURMOUNT-1 trial [5].Recent real-world data of obesity medication usage from Weight Watchers indicates that similar results are being achieved in the community setting, while other real-world studies show much lower weight loss (5.5% TBWL) [22, 23]. This means that there is the possibility that some people may not need to progress to their planned MBS, although there is currently no evidence on how to predict who might fall into the category, the durability of the effect or the relative risks and benefits of continuing with pharmacotherapy instead of proceeding with MBS. How long patients are prepared to continue with chronic OMM, even when they have had more than 20% weight loss remains contentious. Overall, adherence with OMM is low after 12 months [24, 25], suggesting that despite good weight loss, many patients may need additional therapies, such as endoscopic interventions or MBS. If a person who has achieved good weight loss with OMM then wishes to consider MBS, the weight prior to commencement of OMM should be considered the weight that defines surgical eligibility [7].
4. *Recurrent weight gain*: Whilst some degree of recurrent weight gain is to be expected after any MBS procedure, around 15–20% of people will experience weight recurrence that impacts their health and necessitates reintervention, which has usually meant re-operation [20]. Endoscopic interventions, such as transoral outlet reduction, have been used to manage weight regain after Roux-en-Y gastric bypass, with promising outcomes up to 12 months [26], although data from high-quality longer term studies is lacking [27]. It was noted that there is currently limited evidence regarding the best dosing, best schedule, and potential adverse effects of adding OMM in this situation, but thus far no major challenges have emerged from RCT data or real world data [11, 12, 28, 29].
5. *Sub-optimal clinical or health response to MBS*. Whilst 85–90% of people with the disease of obesity who undergo a MBS can expect ≥ 20% TBWL [30], those who have a suboptimal response have had few options other than revisional surgery to support improvement in outcomes. Whilst OMM or endoscopic therapies may be helpful in this setting, there is currently limited literature to support their use, as noted previously. There is also limited evidence regarding the timing of starting adjuvant therapies in this group of patients. Weight loss three months post-MBS can predict that a person who has undergone MBS is on a sub-optimal weight loss trajectory [31]. However, there is currently no evidence to support intervening at this point. There is also currently no reliable way to predict a sub-optimal response to MBS prior to surgery.
6. *Unwanted Side Effects of MBS*. The most common side effect that leads to conversion, reversal, or revisional surgery is gastroesophageal reflux disease (GERD) after sleeve gastrectomy. There is no evidence that OMM will improve GERD following MBS and given their effect on delaying gastric emptying, it is possible that GLP-1RA may worsen GERD. Endoscopic therapies such the Stretta^®^ procedure [32], anti-reflux mucosectomy [33] and endoscopic sleeve refashioning [34] have all been proposed to assist with the management of GERD after sleeve gastrectomy, however, there is currently limited evidence of sustained benefit.

Postprandial hypoglycemia is a challenging complication of MBS for which effective and tolerable pharmacological treatments are limited. While increased post-prandial release of endogenous GLP-1 after MBS likely contributes to its pathophysiology, there is evidence suggesting that GLP-1RAs could reduce the number of postprandial hypoglycemic episodes. The potential of this treatment approach requires further investigation.

**Table:** 

Key themes identified as requiring more evidence through expert discussion:
• Use of OMM or reversible endoscopic therapies prior to referral for MBS.
• Use of OMM or reversible endoscopic therapies as a weight loss and health improvement tool in preparation for MBS.
• Use of OMM or endoscopic therapies after MBS.
◦ Recurrent weight gain.
◦ Timing of intervention with OM or endoscopic therapies following a sub-optimal response to MBS.
◦ Endoscopic therapies or OMM for unwanted side effects of MBS.

### Research Questions

The review of the IFSO consensus statement, assessment of the current literature, and expert discussion resulted in the group identifying several areas with knowledge gaps.

Five key research questions were developed when considering how these gaps might be addressed.

1. How do MBS, OMM, and endoscopic procedures such as endoscopic sleeve gastrectomy (ESG) when used as single-line therapies compare in terms of efficacy and safety in the short- and long-term?
2. Does treatment with OMM or reversible endoscopic obesity therapies (such as intragastric balloon) prior to MBS enhance the efficacy or safety of various MBS procedures?
3. Does the use of OMM after MBS increase efficacy, optimize maintenance, and/or minimize weight loss relapse?
4. What is the best pathway for those with a sub-optimal clinical response to MBS?
5. Can MBS or endoscopic interventions be used for long-term weight maintenance in individuals who have lost weight with OMM and do not wish to continue the medications?

### Potential Trial Methodologies

Traditional RCTs are the acknowledged gold standard study design to examine causal relationships between interventions and outcomes. The continuing rapid expansion in the range of endoscopic and pharmacotherapies for obesity poses a challenge for the design of traditional RCTs to address some of the identified knowledge gaps. Additionally, given the high cost of RCTs and the treatment options under investigation, it is likely that a coalition of funders would be required. This reflects the transdisciplinary approach needed to address many of the unanswered questions in the combination therapies space.

Where these challenges cannot be adequately met by traditional RCTs, alternative prospective study designs that may be more suited to addressing the key research questions in an agile and dynamic way to inform timely practice guidance also need to be considered (Table 3). We have focused on methodologies that utilise prospective data as there is limited retrospective data currently available on these new OMM:

**Table 3:** Potential Pragmatic Approaches to Research Questions Developed by the Expert Group [35–38]

Trial type	Description	Advantages	Disadvantages
Platform Trials	- Multiple treatments evaluated within a single master protocol. - Allows adaptive design for adding/removing arms. - Common control group used.	- Efficient use of resources (shared control arm). - Can evaluate multiple interventions simultaneously. - Adaptability to new data. - Faster trial completion for individual arms.	- Complex design and statistical analyses. - Requires significant upfront planning. - Regulatory challenges.
In Registry RCT	- Randomized controlled trial embedded within a clinical registry. - Utilizes real-world data (RWD) infrastructure for recruitment and follow-up. - Includes randomization for causal inference.	- Cost-effective due to existing infrastructure. - Reflects real-world practice. - Streamlined data collection. - Easier recruitment in registry populations.	- Limited to data captured by the registry. - Potential for incomplete or inconsistent data. - Registry quality affects validity.
Prospective Registry Studies	- Observational study using a registry to collect data on predefined cohorts prospectively. - No randomization.	- Captures real-world evidence. - Useful for long-term safety and effectiveness outcomes. - Less expensive than RCTs.	- No randomization, leading to potential bias. - Confounding variables harder to control. - Limited to observational data.

- Platform trials take an adaptive, prospective, disease-focussed (rather than treatment focussed) approach which potentially compares multiple simultaneous or sequential interventions, often using a Bayesian statistical approach, where hypotheses (or treatment strategies) are refined on the basis of new information [35]. Platform trials have high upfront set up costs, but can enable multiple research questions to be addressed, and can be readily adapted as new treatments and technologies emerge [36].
- Pragmatic RCTs utilizing existing MBS registry platforms create efficiency and significantly reduce trial costs if participants were randomised to treatments that were already being funded and provided in established healthcare systems, rather than the costs of the treatment under investigation being met by the trial funders [37].
- Prospective registry studies – document the real-world experience of the treatments, but with agreed and consistent methodological approaches, outcomes and assessment intervals [38].

### Key Issues to be Addressed Prior to Trial Design

Critical to the successful completion of any trial is a clear understanding of the purpose of the trial, the characteristics of the target cohort(s) and clear definitions of appropriate outcome measures. An emerging area in obesity management is identifying clearly what level of weight loss we should be aiming for if health improvement is our primary concern.

#### Prioritisation

The current knowledge gaps will need to be identified and prioritised prior to the commencement of any trial. Prioritisation should include the perspective of various stakeholders and, importantly, the patient’s voice. The patient perspective should be included at all stages of the trial: in the design phase through surveys, consultations, panels and advisory groups; during the trial through patient reported outcomes and experience measures, focus groups and oversite governance committees; in the write up phase as part of the authorship group and in post-trial assessments through focus groups, alumni surveys and impact assessments [39].

#### Appropriate Test Cohort

A test cohort should ideally offer the opportunity to test the null hypothesis with the minimum number of patients. Cohorts with the opportunity to see the most significant magnitude of the effect may include those with BMI ≥ 50 kg/m^2^, those with high healthcare resource usage, and those with multiple diagnoses that could be improved with weight loss.

#### Outcome Measures

Outcome measures would ideally include not only changes in weight and body composition but also patient-reported outcomes and experience measures, as well as health improvement, response rates and biobanking. These could be based upon core sets of outcome measures that have previously been validated and published [40–42].

#### Defining Treatment Targets

For many chronic conditions, such as T2D and hypertension, a fixed general treatment target is set based on an overall balance of benefits and risks, although the risk of complications is continuous. For example, for adults with diabetes, the general target is an HbA1c < 7.0%, even though the onset and progression of microvascular complications is lower with further reductions in blood glucose levels. Hence, a higher or lower HbA1c target may be appropriate for an individual, depending on their risks of hypoglycaemia, complications, life expectancy, and comorbidities.

Many health risks associated with obesity are similarly on a continuum, but there are currently no widely accepted general targets for treatment. Common approaches include aiming for an arbitrary BMI (fixed) target or an amount of weight loss (change from baseline).

After MBS, a BMI of 25 kg/m^2^ is an implied target, as weight outcomes have historically been defined in terms of “percent excess BMI loss” or “percent excess weight loss” in relation to a reference BMI of 25 kg/m^2^. However, for individuals with obesity, evidence that reducing weight to a BMI of 25 kg/m^2^ is linked with clear health benefits or risk reductions to the level of those without a history of obesity is lacking.

Due to limitations in BMI as a measure of adiposity at an individual level, it has been proposed that at least one anthropometric criterion (e.g., waist circumference, waist-to-hip ratio, or waist-to-height ratio) in addition to BMI, or direct measurement of body fat, where available, should be used to confirm whether a person has excess adiposity, using validated methods and cutoff points appropriate to age, gender, and ethnicity [43]. How we optimally define excess body weight remains to be determined.

In the absence of defined cut-offs for measures of adiposity predictive of individual health risks, obesity management guidelines have often recommended the loss of a proportion of body weight (percent weight loss), regardless of starting weight. These goals are typically below the mean total body weight losses expected from bariatric surgeries and are primarily based on health improvements demonstrated in clinical trials of lifestyle interventions [44, 45].

An emerging concept for defining an end target for disease intervention is the incorporation of the particular clinical feature or biomarker used as the threshold for defining the need for treatment. For people with type 2 diabetes, for example, this might be achieving an HbA1c below the diagnosis threshold of 6.5%; alternatively, if declining kidney function is the main impetus for treatment, then stabilisation of kidney function might be the selected target.

A recent international consensus defined the concept of clinical obesity as excess adiposity causing alterations in the function of tissues, organs, or the entire individual [43]. Thus, for people with “clinical obesity”, a potential target could include reversal of organ or tissue dysfunction caused by excess adipose tissue [44, 45].

Defining meaningful targets is an important avenue for future research and much work needs to be done to understand the role of body composition and health outcomes on quality and quantity of life after obesity treatment. Evidence emerging from real world data in primary care suggest that patients who lose weight and achieve a BMI < 27 kg/m^2^ have greater improvements of mechanical complications of obesity compared to those who lose weight but remain above these thresholds [46, 47] There is also evidence suggesting the greater the reduction in height: waist ratio, the more metabolic benefit [48]. These data need further validation and appropriate target height: waist ratios still need to be defined, however, they could be pragmatic treatment targets able to be tested in more robust interventional trials.

## Conclusion

Through a multi- and inter-disciplinary expert review of the literature and discussion, multiple areas where there is insufficient evidence to guide practice on combined obesity therapies have been identified, generating important research questions. There are several methodological options to address these questions, and ideally, a trial design that can adapt to emerging treatments and to shifting clinical and patient requirements and expectations should be utilised. Further work is needed to prioritize research questions and identify relevant outcome measures. We will likely need to look beyond conventional RCTs to answer some of these questions.

## Data Availability

No datasets were generated or analysed during the current study.
